# Awareness and Behaviors Regarding COVID-19 among Albanian Undergraduates

**DOI:** 10.3390/bs11040045

**Published:** 2021-03-31

**Authors:** Francesca Gallè, Arjeta Veshi, Elita Anna Sabella, Morena Çitozi, Giovanna Da Molin, Stefano Ferracuti, Giorgio Liguori, Giovanni Battista Orsi, Christian Napoli, Christian Napoli

**Affiliations:** 1 Department of Movement Sciences and Wellbeing, University of Naples “Parthenope”, Via Medina n. 40, 80133 Naples, Italy; giorgio.liguori@uniparthenope.it; 2 Fakulteti i Shkencave Psikologjike, Sociale dhe Politike, Universiteti Mesdhetar i Shqipërisë, Bulevardi Gjergj Fishta 52, 1023 Tirana, Albania; arjeta.veshi@umsh.edu.al; 3 Inter-University Research Centre “Population, Environment and Health”, University of Bari Aldo Moro, Piazza Umberto I, 1, 70121 Bari, Italy; elita.sabella@uniba.it (E.A.S.); giovanna.damolin@uniba.it (G.D.M.); 4 Fakulteti i Shkencave Juridike dhe Marrëdhënieve Ndërkombëtare, Universiteti Mesdhetar i Shqipërisë, Bulevardi Gjergj Fishta 52, 1023 Tirana, Albania; morena.citozi@umsh.edu.al; 5 Department of Human Neurosciences, Sapienza University of Rome, Piazzale Aldo Moro 5, 00185 Rome, Italy; stefano.ferracuti@uniroma1.it; 6 Department of Public Health and Infectious Diseases, Sapienza University of Rome, Piazzale Aldo Moro 5, 00185 Rome, Italy; giovanni.orsi@uniroma1.it; 7 Department of Computer Control and Management Engineering “Antonio Ruberti”, Sapienza University of Rome, Via Ariosto 25, 00185 Rome, Italy; cnapoli@diag.uniroma.it or; 8 Department of Medical Surgical Sciences and Translational Medicine, Sapienza University of Rome, Via di Grottarossa 1035/1039, 00189 Rome, Italy; christian.napoli@uniroma1.it

**Keywords:** COVID-19, knowledge, behaviors, lockdown, undergraduates

## Abstract

The coronavirus disease 2019 (COVID-19) pandemic has led to the adoption of restriction measures that have had notable consequences on the health and wellbeing of individuals. This survey was carried out on a sample of 905 Albanian undergraduates to assess their knowledge about COVID-19 and their health-related behaviors and communication changes during lockdown. The students were invited to complete a pre-validated questionnaire during lessons. The results show that the majority of the surveyed students were able to answer correctly about the main epidemiological features of the disease. The level of knowledge was proven to be proportional to the students’ education level (enrollment year and age). No considerable relationship emerged with respect to diet or smoking. On the other hand, a relevant portion of the sample (37.6%) declared decreased physical activity (PA). Finally, a notable increase in the intensity and frequency of communication with their peers and with their parents was declared by 34.7% and 50.8% of the sample, respectively. Lifestyle variables were found to be mutually related, as were communication outcomes. The participants showed a satisfactory awareness regarding the COVID-19 epidemic. However, the registered reduction in PA may represent a public health issue and should be addressed with adequate policies. The use of electronic media seems to have increased communication habits in this population group.

## 1. Introduction

Since late 2019, severe acute respiratory syndrome coronavirus 2 (SARS-CoV-2) has spread worldwide, causing a serious pandemic. As of 22 January 2021, more than 2 million global deaths due to coronavirus disease 2019 (COVID-19) have been reported [1].

Specific treatments and/or vaccines were not available at the beginning of the pandemic; therefore, in order to contain the spread of the virus many countries enforced different control measures, such as contact tracing for SARS-CoV-2 among ill patients and relatives due to the demonstrated effectiveness of screening for infectious diseases in at-risk populations [2,3]. Moreover, personal contact and gatherings have been limited, e.g., by limiting or forbidding access to workplaces or educational institutions, up to the enforcement of nationwide lockdowns [1,4]. Such measures have had notable impacts on the socioeconomic systems of many countries, as well as detrimental effects on the health and wellbeing of individuals [5,6,7,8].

During the first phases of the pandemic, the control of the spread of the virus relied mainly on the ability of citizens to maintain social distancing, respect isolation, wear face masks and practice adequate hand hygiene. An individual’s ability to implement such prevention measures is based on their understanding of COVID-19 etiopathogenesis and transmission. The literature shows that higher levels of knowledge regarding COVID-19 are associated with a lower likelihood of attitudes and practices that may have negative effects in the pandemic management [9]. Similarly, age, gender and education level seem to be paramount factors related to knowledge and risk perception, therefore resulting in the adoption of prevention measures, and also preparedness for disasters caused by COVID-19 [10]. 

With almost three million inhabitants and a life expectancy at birth of 78 years, Albania has a gross domestic product (GDP) greater than 15 thousand billion USD, which has significantly increased over the last 20 years. In terms of the distribution of inequality, Albania shows a Gini index (33.32) in line with other European countries, where a Gini index of 0 represents perfect equality, while an index of 100 implies perfect inequality [11,12]. The percentage of illiterate inhabitants (≥15 years old) is around 1.5%, and the gross enrollment ratio for tertiary education (≥18 years) is around 60% [11]. In Albania, the epidemic started in March 2020, and about 70,000 cases and 1300 deaths have been reported as of 22 January 2021 [1]. Under these conditions, Albanian teaching institutions, including the academic community, continued their activities by means of online learning platforms. Information regarding adaptation to and perception of online learning during the COVID-19 pandemic by Albanian university students, and also regarding their mental health conditions, has already been described [13,14]. However, no data are available so far on COVID-19 awareness in the first phase of the epidemic, nor on lifestyle changes adopted during lockdown among Albanian undergraduates. Since the first step in the process of pandemic disaster management is the assessment of the population’s basic level of knowledge about the emergency [10], this information gap should be filled. Moreover, with Albania being a representative country of southeast Europe, the scientific evidence coming from this territory may contribute to completing a continent-wide picture of this issue.

In this context, a sample of Albanian undergraduates was surveyed in order to evaluate (i) their level of knowledge about the epidemiological characteristics of and preventive measures against COVID-19 in the first few months of the epidemic; (ii) the lifestyles adopted during lockdown; (iii) their opinions about the online learning provided during the emergency period and the related changes in communication.

## 2. Materials and Methods

This cross-sectional study was performed during the last two weeks of April 2020 among Albanian undergraduate students from universities in Tirana. During that period, due to the COVID-19 epidemic, the selected universities were already providing lectures via the internet. 

For this study, the adopted inclusion criteria limited the survey participation to undergraduate students regularly attending lectures in one of the selected universities. Convenience sampling was performed by inviting students attending remote lessons to voluntarily participate in the survey by responding to an online questionnaire. Due to the persistent emergency and the related difficulties in the survey enrollment procedures, it was not possible to randomize or stratify the sampling, as in similar studies [12,15]. The Web-based survey was sent via email and remained open until 30 April 2020. 

Since the whole target population of Albanian undergraduates was 127,477 people, a sample of at least 383 individuals would have been required to explore the selected variables assuming a response proportion of 50%, within 95% confidence level and a 5% margin of error, as previously reported [16]. The study was previously performed among Italian undergraduates [15]. The questionnaire, accurately validated during the previous study [15], included three sections (Appendix A). 

The first section of the questionnaire was focused on demographic information (gender, age, degree, year of enrollment); students were also asked to report if they knew someone who was affected by COVID-19.

The second section included questions regarding the undergraduates’ knowledge about COVID-19 and related control measures. Students were asked to evaluate their own level of knowledge on the epidemic (e.g., the causative agent, the main transmission route, diffusion, etc.), the most effective preventive measures and therapy, and the institutions managing the emergency.

The third section aimed to investigate the lifestyle, such as current diet, smoking and physical activity (PA), of undergraduates during lockdown as compared with the pre-epidemic conditions. In addition, we included questions regarding their frequency of attendance to lessons, their opinion about the functioning of online courses, and possible changes that had occurred in their communication with other students and with their parents.

The Cronbach’s alpha value achieved for the present investigation was 0.79, showing a satisfactory level of internal consistency [17]. 

The study respected the World Medical Association Declaration of Helsinki. No experiments on biological human samples nor research on identifiable human data were performed. The study protocol was approved by the competent committee of the Inter University Research Centre “Population, environment and health” (CIRPAS) (approval number 1603_2020).

### Statistical Analyses

We also conducted a descriptive analysis of the undergraduates’ answers in relation to their demographic characteristics. Continuous outcomes were expressed as mean values ± standard deviation (SD). Knowledge and behaviors were reported as the number and percentage of respondents. A Pearson/Spearman correlation analysis was carried out to highlight possible relationships between the variables explored. In particular, age, gender (0 = male, 1 = female) and attended year of university education were included as demographic variables. With regard to the second section of the questionnaire, the level of knowledge was expressed as the total number of correct answers (range 0–13); multiple-answer questions were considered correct when at least one correct answer was chosen by the student. Lifestyle and communication variables were categorized as worsened (0), maintained (1) and improved (2). 

The software IBM SPSS version 26 for Windows (IBM Corp., Armonk, NY, USA) was used for the analysis.

## 3. Results

A total of 905 students completed the questionnaire. The response rate was 30.3% (905 out of 2990 invitations sent). The students reported attending four different universities located in the city of Tirana. Table 1 shows the main characteristics of the sample.

Answers regarding undergraduates’ knowledge about COVID-19 are summarized in Table 2.

The majority of the sample defined their own level of knowledge about the epidemic as “good”. About three quarters of the sample considered the current epidemic similar to seasonal flu. 

Almost all the participants (96.5%) identified a virus as the causative agent of the epidemic and indicated air as the main route of transmission (77.6%). The whole population was indicated as the target of the disease by the majority of the sample (95.1%). The avoidance of close contact (90.6%), handwashing (76.6%), the use of facial masks (66.6%) and gloves (64.2%), and the disinfection of surfaces (61.7%) were identified as the main protective measures against COVID-19 transmission. The majority of the sample declared that the disease cannot be treated with antibiotics (70.1%), nor, at that time, prevented by a vaccine (65.4%); meanwhile, less than half of the participants were confident in the availability of a specific drug for treatment (48.6%).

The majority of the sample identified the whole Albanian territory as involved in the epidemic (67%), a reduction in infected people as the reason for the lockdown (72.2%), and people living in high-risk areas as the target for diagnostic tests (74.4%). The responsibility for the management of the health emergency was attributed mainly to the Prime Minister (40.3%). Almost the entire sample was aware of a number of reported cases near to 1000 at the time of the survey (97.7%).

Table 3 reports the answers of participants regarding the lifestyles adopted during the lockdown. As for diet, basically no change in their habits were reported (41.9%). The majority of the participants declared themselves non-smokers both before and during the lockdown (81.3%), while 13.4% continued to smoke. Interestingly, 4.4% of former smokers had quit smoking. As for PA, about 37.8% of the respondents had not changed their usual habits, while 37.6% had decreased their habitual level of PA. 

Table 4 shows the statements and judgments collected about the communication during lockdown.

Overall, 97% of the respondents reported attending online lectures, and 44.1% considered the online system to be working well. The reasons adduced to malfunctioning were mainly linked to personal devices. About half of participants reported unchanged levels of communication with their peers (52.4%), with an increase declared by another 34.7% of them. Interestingly, half of the sample (50.8%) reported an increase in communication with their parents.

As for correlation analyses, the level of knowledge was found to be proportional to age (r = 0.102, *p* = 0.002) and years of university education (r = 0.137, *p* < 0.001), as well as inversely proportional to changes in communications with other students (Rho = −0.086, *p* = 0.010). The improvement of dietary habits was found to be positively related to years of education (Rho = 0.066, *p* = 0.049), as well as improvements in smoking habits (Rho = 0.099, *p* = 0.003) and in PA (Rho = 0.147, *p* < 0.001). Communication among students was found to be directly related to communication with parents (Rho = 0.188, *p* < 0.001).

## 4. Discussion

The present study reports the results of a survey regarding knowledge about COVID-19 and related behaviors among undergraduates in the first phase of the pandemic in Albania. To the best of our knowledge, this is the first contribution to the assessment of the Albanian population’s awareness about COVID-19 risks and lifestyle adopted during lockdown.

Although the majority of the sample was aware of the main epidemiological features of the disease, there is space for further knowledge improvement. This is in line with previous findings obtained among undergraduates in both European countries (EU) and non-EU countries [15,16]. However, several critical issues have emerged. Firstly, more than half of the participants believed that the virus is also transmitted by non-airborne routes. Secondly, more than one third of the undergraduates did not consider either facial masks or the disinfection of surfaces to be effective protective measures. This latter finding confirms previous study and highlights that we need to pay attention to these issues in public education, and also beyond the specific case of pandemics [16]. It should be noted that, at the time of survey, facial masks for the general population were not yet recommended at community level; therefore, the awareness regarding the use of such protective equipment could have improved since. 

Almost half of the surveyed students declared that a specific drug was available to treat COVID-19, although this is an incorrect belief that could tamper with the implementation of preventive measures, since it can cause or evolve into a reduced perception of the pandemic’s severity. In fact, a significant correlation has been demonstrated between self-reported preventive behaviors and lack of risk perception [16]. 

Even though the whole country was correctly considered to be involved in the epidemic, the majority of the respondents indicated that diagnostic tests should be performed only among people from areas at high risk of transmission. This latter result is not consistent with other experiences, where a greater awareness concerning diagnostic and screening methods was shown among undergraduates [15]. 

These misunderstandings may have been generated by misleading news diffused through media communication services, as reported in the previous SARS epidemic [18]. Therefore, especially in the case of an emergency, it is important to put in place, as soon as possible, communication strategies based on a cross-disciplinary perspective accessible to the entire population [19]. For this purpose, scientific evidence demonstrates that, regarding the pandemic and related protective measures, awareness campaigns should be customized for specific target segments of the population [20]. Although our questionnaire does not investigate the students’ sources of information, it should be considered that, due to the increased amount of time spent at home during lockdown, they had a greater opportunity to watch TV and surf the Web [15]. 

Moreover, in this study a small percentage of students (6.1%) reported a case of COVID-19 among their contacts as compared with previous experience [15]. It has been found that having relatives or acquaintances infected with COVID-19 is a risk factor for increased anxiety among college students [15], and therefore, this can explain an increased need to improve their knowledge about the disease and its containment.

Concerning lifestyle, no remarkable changes were registered regarding smoking, while a worsening diet and a decrease in PA were acknowledged by a relevant portion of the sample. These results are consistent with previous findings coming from both EU countries during the COVID-19 pandemic, and other contexts [7,15,21]. The role of factors such as nutrition and PA in maintaining health and wellbeing is central. In particular, since a dose–response relationship between PA performed before infection and a decrease in the incidence, duration or severity of acute respiratory tract infections has been demonstrated [22], empowering people to actively preserve their health should be stressed [23]. 

In the present study, we also sought to assess how the use of electronic media during lockdown was perceived by students and how it changed their usual interpersonal communication. While most of the surveyed students attended lessons online as those held in-person before the pandemic outbreak, more than half of the participants indicated a general dissatisfaction regarding the online didactics as provided by their university, answering “not” or “not completely” when asked whether the online courses were working for them. However, such dissatisfaction was mainly reported as caused by issues related to students’ personal devices. In line with these findings, a study performed by Xhelili et al. among Albanian undergraduates reported a high percentage of undergraduates who were unsatisfied with online learning, mainly due to a lack of familiarity with technology [13]. While the pandemic continues, and since restriction measures still have to be applied, this issue should be considered in order to support Albanian university students adapting to the new learning modalities.

A considerable increase in communication with both peers and parents was reported. In this regard, Boniel-Nissim et al. found that an “optimal” frequency of electronic communication with friends might be associated with life satisfaction; on the other hand, it has also been devised that if this frequency exceeds a certain threshold, the consequences may be negative [24]. Unfortunately, we did not explore life satisfaction among the participants.

Regarding the correlation analyses, we found that the level of knowledge was proportional to age and years of education, confirming the role of these variables in determining a better ability to discern correct information on health [25]. Interestingly, the level of knowledge about the epidemic was inversely related to communication with other colleagues. This finding might highlight the spread of incorrect information in the students’ social communities and should be further explored.

Changes in diet habits were directly related to changes in smoking and PA, in line with previous studies [26,27,28]. This confirms that, also for Albanian undergraduates, the improvement of one lifestyle aspect can also positively influence overall healthier behavior. We can conclude that lockdown, in some cases, has been an opportunity to improve lifestyle.

Finally, it emerged that changes in communication among colleagues was also related to similar changes in communication with parents. This finding could be considered a consequence of the popularity gained by multiple electronic communication platforms, which has been followed by increased usage for communication among individuals [29]. These latter circumstances have likely been triggered by the movement restrictions and consequent need to remain in contact with relatives and parents [30]. 

The effects of the COVID-19 pandemic and quarantine on the psychological wellbeing of individuals were examined in previous studies in Albania and other EU countries [12,14,31], demonstrating that the pandemic acted as a stressor with important differences related to gender, age and country. In particular, undergraduates’ psychological health was substantially affected by the COVID-19 pandemic, and academic and relational changes were the major sources of stress [31]. At the same time, it seems that parent–child discussion is a protective factor for mental health [32]. Therefore, these aspects should be considered for their potential role as psychological factors associated with coping strategies during the pandemic. Unfortunately, our questionnaire was aimed only at assessing the undergraduates’ perception and not at investigating the features of communication they experienced during lockdown. Further studies are needed to highlight which factors are improving or worsening communication with peers and parents during the pandemic. 

This study has some limitations. First, the sample does not completely represent the Albanian undergraduate nor young adult population. Moreover, due to sample characteristics, it was not possible to perform a comparison between genders or different areas of study. Furthermore, in order to avoid an oversized questionnaire, socioeconomic level, sources of information, psychological distress and communication features were not investigated. Therefore, it was not possible to characterize predictors of knowledge, lifestyles and communication outcomes. Finally, while the study should be considered preliminary research, it provided important information about knowledge and behaviors of undergraduates during the COVID-19 epidemic in Albania, contributing to a more complete map of the level of knowledge of undergraduates during the current emergency in the European region.

## 5. Conclusions

In spite of some limitations, this study provides an assessment of undergraduates’ knowledge and behaviors related to the COVID-19 pandemic in Albania. The findings can be used to improve the effectiveness of information and pandemic control campaigns by means of more accurate targeting.

With regard to the disease and its prevention, Albanian undergraduates showed an acceptable level of knowledge. Nevertheless, more attention should be paid to some issues, such as hand hygiene, use of facial masks and surface hygiene. 

With regard to lifestyles, a notable part of the sample reported worsening diet and decreasing PA. Therefore, the promotion of PA during a pandemic is essential to counteract the possible negative effects of a sedentary lifestyle on people’s health. At the same time, promoting PA during the non-pandemic period may also have positive effects in the event of a lockdown [7].

With regard to online lessons and communication features, our results suggest that technology should be integrated gradually into Albanian education and social relationships. In this emergency, considerable support should be provided by academic staff [13]. At the same time, improving communication, also by means of online media, with peers and parents may have positive effects on mental health too. 

Overall, it is confirmed that communication campaigns aimed at educating people towards preventive measures are crucial in the management of a pandemic, in order to prepare citizens for the immediate and long-term health emergency and help them to manage risk and reduce anxiety and fear [19,33]. Effective communication strategies should be targeted at different communities or community groups on the basis of their demographic and sociocultural characteristics, and addressed at filling specific knowledge gaps [10,19,33].

## Figures and Tables

**Table 1:** Characteristics of the sample.

Variable	
Age	
*mean ± SD*	21.2 ± 3.7
Gender	
*n (%)*	
male	234 (25.9)
female	671 (74.1)
Year of the course	
*n (%)*	
First	322 (35.6)
Second	190 (21.0)
Thrid	200 (22.1)
Fourth	127 (14.0)
Fifth	35 (3.9)
Out of course	31 (3.4)
Having contacts affected by COVID-19	
*n (%)*	
Yes	55 (6.1)
No	850 (93.9)

**Table 2:** Answers provided by students regarding COVID-19.

Question	n of Respondents (%)
**Your knowledge of the COVID-19 epidemic is:**	
excellent	337 (37.2)
good	419 (46.3)
medium	145 (16)
low	4 (0.4)
**May the new epidemic be considered similar to that of the seasonal flu?**	
yes	665 (73.5)
no	240 (26.5)
**The cause of COVID-19 is a**	
virus	873 (96.5)
bacterium	30 (3.3)
parasite	2 (0.2)
**The main transmission route of the disease is:**	
air	702 (77.6) ^1^
water	132 (14.6)
food	185 (20.4)
blood transfusion	239 (26.4)
sexual intercourse	230 (25.4)
**What is the target population of COVID-19?**	
only older people	43 (4.8)
only children	1 (0.1)
everyone	861 (95.1)
**The most effective protective measure against SARS-CoV-2 is:**	
handwashing	693 (76.6) ^1^
facial masks	603 (66.6)
gloves	581 (64.2)
protective glasses	109 (12.0)
washing fruit and vegetables	397 (43.9)
condom use	97 (10.7)
disinfection of surfaces	558 (61.7)
avoiding close contact	820 (90.6)
**Is it possible to treat COVID-19 with antibiotics?**	
yes	271 (29.9)
no	634 (70.1)
**Is there a specific drug to treat COVID-19?**	
yes	440 (48.6)
no	465 (51.4)
**Is there a vaccine to prevent COVID-19?**	
yes	313 (34.6)
no	592 (65.4)
**Which area of the Albanian territory has been involved in the epidemic?**	
Northern cities	54 (6.0)
Central cities	238 (26.3)
Southern cities	7 (0.8)
All areas	606 (67.0)
**The lockdown has been adopted in order to:**	
reduce the severity of the disease	234 (25.9) ^1^
decrease the number of infected people	653 (72.2)
enhance people’s immune status	22 (2.4)
reduce the burden of patients	254 (28.1)
**Which categories undergo diagnostic tests?**	
patients with acute symptoms	42 (4.6)
patients with acute symptoms and their contacts	178 (19.7)
people who live in areas at high risk	673 (74.4)
only healthcare personnel	12 (1.3)
**Which institution is managing the health emergency in Albania?**	
Ministry of Health and Social Protection	266 (29.4)
Institute of Public Health	260 (28.7)
Prime Minister	365 (40.3)
Civil Protection	9 (1.0)
Municipalities	5 (0.6)
**How many people have been affected by COVID-19 in Albania, so far?**	
1000	884 (97.7)
10,000	21 (2.3)

^1^ Multiple-answer question.

**Table 3:** Lifestyle adopted by participants during lockdown.

Behavior	Number of Respondents (%)
**Diet**	
same as before	379 (41.9)
less or better than before	267 (29.5)
more or worse than before	259 (28.6)
**Smoking**	
not before nor currently	736 (81.3)
not before but now yes	8 (0.9)
yes before and now	121 (13.4)
yes before but not now	40 (4.4)
**Physical activity**	
decreased	340 (37.6)
increased	223 (24.6)
active as before	170 (18.8)
inactive as before	172 (19.0)

**Table 4:** Statements and judgments of participants about online lessons and communication features during lockdown.

Question	Number of Respondents (%)
**Are you attending online lessons?**	
Yes, as much as before in person	560 (61.9)
Not as much as before in person, but enough	250 (27.6)
Not as much as before in person, and not enough	68 (7.5)
Absent	27 (3.0)
**Are the online courses working well?**	
yes	399 (44.1)
no	83 (9.2)
not completely	423 (46.7)
**If not, what are the reasons for malfunctioning?**	
Teachers’ capability	11 (13.3)
Personal equipment	64 (77.1)
University video communication platform	8 (9.6)
**The communication with your colleagues is:**	
the same as before	474 (52.4)
increased	314 (34.7)
decreased	117 (12.9)
**The communication with your parents is:**	
the same as before	395 (43.6)
increased	460 (50.8)
decreased	50 (5.5)

## Data Availability

Data supporting reported results are available from the authors on request.

## Appendix A. Questionnaire on Undergraduates’ Knowledge about COVID-19 Epidemic

This study is aimed at exploring undergraduates’ knowledge about the COVID-19 epidemic and their lifestyles adopted during lockdown. In line with the General Data Protection Regulations (GDPR) n. 2016/679, your information will be aggregated and treated anonymously. By participating in this study, you declare that you have read and understood these conditions. Thank you for your collaboration.

**Please give your consent for the treatment of your personal information**

yes/no

**Gender:**

Male/Female

**Age:** _____

**University:** ____________________

**Degree Course:** ________________________

**Year of Degree Course:** _______________

**Do you know someone who has been affected by COVID-19?**

yes/no

**Your level of knowledge on the new epidemic is:**

excellent/good/average/low

**The cause of the current pandemic is a:**

virus/bacterium/parasite

**May the new epidemic be considered similar to that of the seasonal flu?**

yes/no

**What is the main transmission route of COVID-19?**

air/water/food/blood transfusion/sexual intercourse

**What is the target population of COVID-19?**

only older people/only children/only adults/everyone

**What is the most effective protective measure against COVID-19?**

handwashing/mask wearing/use of gloves/protective glasses/washing fruit and vegetables/condom use/disinfection of surfaces/avoiding close contacts

**May COVID-19 be treated with the available antibiotics?**

yes/no

**Is there an effective drug for COVID-19 treatment?**

yes/no

**Is there an effective vaccine for COVID-19 prevention?**

yes/no

**Which area of the Albanian territory has been involved in the epidemic?**

Northern cities/Central cities/Southern cities/all areas

**What is the reason for the lockdown?**

reduce the severity of the disease/decrease the number of infected people/enhance the immune status of the population/reduce the burden of patients in the hospitals

**Which categories undergo diagnostic tests for 2019-nCoV detection?**

only patients with acute symptoms/patients with acute symptoms and their contacts/all the people who live in areas at high risk/only health personnel

**Which institution is managing the emergency in Albania?**

Ministry of Health and Social Protection/Institute of Public Health/Prime Minister/Civil Protection/Municipalities

**How many people have been affected by COVID-19 in Albania, so far?**

1000/10,000

**With respect to the time before the lockdown, has your current diet changed?**

I’m eating as before/I’m eating less or better than before/I’m eating more or worse than before

**With respect to the time before the lockdown, has your smoking habit changed?**

I didn’t and I don’t smoke/I didn’t smoke before but now yes/I smoked before and smoke now/I did smoke before but not now

**With respect to the time before the lockdown, has your current physical activity changed?**

it’s increased/it’s decreased/I’m active as before/I’m inactive as before

**Are you currently attending the online lessons provided by your university?**

yes, as much as before in person/not as much as before in person, but enough/not as much as before in person, and not enough/

I’m absent

**Are the online courses working well?**

yes/no/not completely

**If not, what are the reasons for malfunctioning?**

teachers’ capability/personal equipment/university video communication platform

**The communication with your colleagues is:**

the same as before/increased/decreased

**The communication with your parents is:**

the same as before/increased/decreased
